# Critical reflections on post-pandemic dengue surveillance in Quezon city, Philippines

**DOI:** 10.1186/s41182-025-00849-8

**Published:** 2026-01-21

**Authors:** Sheikh Mujahid Rafique, Raza ur Rehman Rana, Irtaza Asghar, Talha Liaquat

**Affiliations:** Shaikh Zayed Postgraduate Medical Institute (SZPGMI), Shaikh Khalifa Bin Zayed Al-Nahyan Medical College, Lahore, Pakistan

## Abstract

This correspondence critically reflects on the recent article by Medina et al. (2025), “Pre- and post-COVID-19 pandemic identification of dengue hotspots and exploration of determinants in Quezon City.” While acknowledging the study’s timely contribution to understanding dengue dynamics in an urban context, the commentary identifies several key limitations. These include the absence of a clear theoretical framework, insufficient adjustment for confounding variables, reliance on a single data type without triangulation, weak translation of findings into actionable policy, and notable omissions in the literature review. Addressing these gaps in future research—through stronger theoretical grounding, rigorous methodological control, integration of multiple data sources, and closer alignment with policy and recent evidence—will enhance the scientific depth and practical relevance of dengue epidemiology.

**Dear Editor,**

We enthusiastically read the article “Pre- and post-COVID-19 pandemic identification of dengue hotspots and exploration of determinants in Quezon City” by Medina et al. (2025) [1] with great interest. This timely work provides important insights into the spatial dynamics of dengue before and after the COVID-19 pandemic, offering valuable contributions to understanding how public health emergencies may reshape endemic disease patterns. Their focus on the interplay between urbanization, public health infrastructure, and disease dynamics highlights critical determinants that remain highly relevant for public health practitioners and policymakers. This work thus contributes meaningfully to ongoing discussions about how local-level analyses can inform regional dengue prevention strategies.

We would like to draw your attention to an important perspective that the study has missed. Firstly, while the study provides useful descriptive insights into dengue hotspot dynamics, its contribution could have been strengthened by more explicit engagement with competing theories or frameworks. A stronger theoretical grounding would allow the authors’ descriptive findings to be more widely generalizable and theoretically anchored. Without this, findings risk being descriptive rather than explanatory, reducing their contribution to cumulative science [2]. The integration of theoretical models allows researchers to place their findings in a broader conceptual landscape. Studies without a guiding framework often lack depth and risk being seen as isolated observations rather than generalizable contributions [3]. The absence of a clear theoretical grounding makes research vulnerable to fragmented interpretations [4]. Secondly, the authors highlight important associations between urban factors and dengue incidence; however, the interpretation of these findings would be further enhanced by addressing potential confounding variables, such as pandemic-related reporting biases, mobility restrictions, and climatic variations. Selection bias and confounding are key risks in observational designs, which can lead to spurious associations if not adequately addressed [5]. Pandemic-related reporting biases act as confounders in dengue surveillance, challenging the reliability of ecological trend analyses [6]. The misinterpretation of associations without controlling for confounding variables exemplifies a recurring epidemiologic fallacy [7].

Thirdly, the study’s reliance on a single dataset provides a clear and focused picture of dengue hotspot trends. Nonetheless, incorporating triangulation through qualitative insights, secondary datasets, or entomological surveillance could have increased the robustness and contextual depth of the conclusions. Reliance on single datasets risks over-simplification; multiple environmental and social data improve interpretation [8]. This triangulated design ensures that quantitative measures are contextualized with qualitative narratives, increasing reliability and applicability [9]. Fourthly, while the article discusses implications, it stops short of demonstrating how findings can be translated into actionable policy or integrated into public health practice. Without clear pathways from research to intervention, surveillance loses effectiveness [10]. Although epidemiological evidence on risk factors is robust, translation into policy action remains uneven and context dependent [11]. To maximize the impact of these findings, future research could go further by demonstrating explicit pathways for linking evidence into policy or practice. Without direct collaboration between researchers and public health stakeholders, epidemiological findings risk remaining descriptive rather than operational [12].

Finally, the literature review synthesizes several important contributions; however, incorporating recent evidence on vectorial capacity, molecular data, and arbovirus seroprevalence would provide a more comprehensive foundation for interpreting the results. Current literature on *Aedes aegypti* highlights ongoing uncertainties in vectorial capacity and viral interactions [13]. Recent literature on vector dynamics remains fragmented, no documented reviews explicitly synthesize current dengue molecular data, highlighting overlooked evidence [14]. There are critical gaps in global arbovirus seroprevalence data, limiting the ability to contextualize recent epidemiological findings [15] .

In summary, while Medina et al. (2025) provide valuable insights into dengue hotspot dynamics in the context of the COVID-19 pandemic. This study advances the discussion on post-pandemic dengue surveillance, and identifying its limitations highlights important directions for future inquiry. The absence of a strong theoretical framework, insufficient control of confounding variables, lack of triangulation, limited policy translation, and notable gaps in the literature review collectively restrict the study’s broader scientific and practical contribution. Future research in this field should be anchored in well-defined theoretical models, employ rigorous strategies to adjust for confounding, and integrate mixed-methods or triangulated approaches to enhance validity. Equally important, translating findings into actionable policy frameworks and ensuring that reviews incorporate the most recent and relevant global evidence will maximize the relevance of such studies for both scholarship and public health practice. Strengthening these aspects will allow future investigations to move beyond descriptive analyses and provide more robust, generalizable, and policy-informing contributions to dengue control.

## Data Availability

No datasets were generated or analysed during the current study.
